# Predicting Anxiety in Individuals with Diabetes: A Comparative Analysis of Machine Learning Algorithms

**DOI:** 10.14341/probl13459

**Published:** 2026-05-20

**Authors:** H. Bourkhime, N. Qarmiche, S. Benmaama, N. Lazar, M. Omari, M. Berraho, N. Tachfouti, S EL Fakir, H. El Ouahabi, N. Otmani

**Affiliations:** Sidi Mohamed Ben Abdellah University; Hassan II University Hospital; Sidi Mohamed Ben Abdellah University; Medical Informatics and Data science Unit, Laboratory of Epidemiology, Clinical Research and Community Health, Faculty of Medicine and Pharmacy of Fez, Sidi Mohamed Ben Abdellah University; Diagnostic center, Hassan II University Hospital; Medical and Pharmaceutical Sciences and Translational Research, Laboratory of Epidemiology and Health Sciences Research, Faculty of Medicine and Pharmacy of Fez, Sidi Mohamed Ben Abdellah University; Sidi Mohamed Ben Abdellah University; Sidi Mohamed Ben Abdellah University; Laboratory of Artificial Intelligence, Data Science and Emerging Systems, National School of Applied Sciences Fez, Sidi Mohamed Ben Abdellah University; Department of Epidemiology, Clinical Research and Community Health, Faculty of Medicine and Pharmacy of Fez, Sidi Mohamed Ben Abdellah University; Hassan II University Hospital; Sidi Mohamed Ben Abdellah University; Diagnostic center, Hassan II University Hospital; Department of Epidemiology, Clinical Research and Community Health, Faculty of Medicine and Pharmacy of Fez, Sidi Mohamed Ben Abdellah University; Hassan II University Hospital; Diagnostic center, Hassan II University Hospital; Department of Endocrinology, Diabetology, Metabolic Diseases and Nutrition, Hassan II University Hospital; Sidi Mohamed Ben Abdellah University; Hassan II University Hospital; Medical Informatics and Data science Unit, Laboratory of Epidemiology, Clinical Research and Community Health, Faculty of Medicine and Pharmacy of Fez, Sidi Mohamed Ben Abdellah University; Diagnostic center, Hassan II University HospitalDiagnostic center, Hassan II University Hospital; Hassan II University Hospital; Sidi Mohamed Ben Abdellah University; Diagnostic center, Hassan II University Hospital; Medical and Pharmaceutical Sciences and Translational Research, Laboratory of Epidemiology and Health Sciences Research, Faculty of Medicine and Pharmacy of Fez, Sidi Mohamed Ben Abdellah University; Department of Epidemiology, Clinical Research and Community Health, Faculty of Medicine and Pharmacy of Fez, Sidi Mohamed Ben Abdellah University; Sidi Mohamed Ben Abdellah University; Hassan II University Hospital; Medical Informatics and Data science Unit, Laboratory of Epidemiology, Clinical Research and Community Health, Faculty of Medicine and Pharmacy of Fez, Sidi Mohamed Ben Abdellah University; Diagnostic center, Hassan II University Hospital

**Keywords:** Diabetes, Anxiety, Machine Learning, prediction, risk, Diabetes, Anxiety, Machine Learning, prediction, risk

## Abstract

Diabetes is a long-term costly burden that increases the vulnerability of individuals to develop anxiety disorders. Consequently, effective management of diabetes anxiety in diabetics can significantly improve overall patient care. This paper presents a comparative analysis of three machine learning algorithms, namely Logistic Regression (LR), Support Vector Machine (SVM), and Decision Tree (DT), in predicting anxiety among diabetics. A Moroccan dataset was utilized, and a grid search approach was employed for hyperparameters tuning.

The findings demonstrate promising results in terms of the algorithms’ performance. The Decision Tree algorithm exhibited the highest accuracy, achieving an impressive 96% in predicting anxiety among diabetics. SVM followed with an accuracy rate of 69%, while LR achieved 61%. These outcomes provide valuable insights for further research endeavors aimed at refining the prediction models.

In conclusion, the study highlights the potential of machine learning algorithms in predicting anxiety disorders among individuals with diabetes. The high accuracy demonstrated by the Decision Tree model suggests its potential as a reliable tool in clinical settings. Further investigations are warranted to validate these results and explore the applicability of these models in real-world scenarios, ultimately enhancing the management and well-being of individuals with diabetes and comorbid anxiety disorders.

## INTRODUCTION

Diabetes mellitus is a prevalent chronic disease that ranks among the top 10 causes of death in adults worldwide [1]. The International Diabetes Federation reports that the global prevalence of diabetes in 2019 was estimated to be 9.3%, affecting 463 million individuals, and it is projected to rise to 10.9%, impacting 700 million people by 2045 [1]. Disturbingly, diabetes claimed the lives of 4 million individuals in 2017 [1]. In the context of Morocco, approximately 2.5 million adults have diabetes, with nearly 50% of them being unaware of their condition [2].

Recent studies have highlighted the intricate relationship between diabetes and anxiety. A study conducted in a Moroccan region in 2021 found that the prevalence of anxiety among individuals with diabetes was 29.6% [3]. Recognizing the significance of addressing the psychological aspect of diabetes management is essential, as anxiety can exacerbate diabetes-related complications, hinder effective disease management, and adversely affect overall patient well-being [4–6].

Machine learning (ML) models have emerged as efficient tools for tackling healthcare challenges, enabling disease prediction and facilitating informed decision-making regarding patient management, thus improving healthcare services [7-8]. In this context, developing an accurate ML model for predicting anxiety in individuals with diabetes is crucial for several reasons.

Integrating anxiety prediction into routine care will enable healthcare providers to identify at-risk patients earlier, allowing for timely psychological interventions that prevent anxiety symptoms from escalating. This approach will also improve personalized treatment plans by addressing both the physical and mental health needs of diabetic patients [7–8].

By leveraging the power of ML, this study aims to develop a model for predicting anxiety in individuals with diabetes. To identify the algorithm that exhibits optimal performance, a comparative analysis of three ML algorithms was conducted, paving the way for future investigations in this domain. This approach not only supports the holistic care of diabetic patients but also underscores the potential of advanced technologies in revolutionizing endocrinological practice.

## RELATED WORKS

Generally, in the field of mental health research, the prediction of anxiety disorders has garnered significant attention. Previous studies have employed various machine learning algorithms, including Artificial Neural Networks (ANNs) [9], Naïve Bayes (NB)[10], Bayes Network (BN)[10], Support Vector Machine (SVM) [9], Decision trees (DTs) [9], Local Nearest Neighbor (LNN) [10], Multilayer Perceptron (MLP) [10], Radial Basis Function Network (RBFN) [10], Linear Regression (LR) [9], Random Forest (RF) [10], and J48 [10]. These algorithms have been applied to diverse datasets, encompassing the online DASS42 scale [10], electronic health records [11], and other relevant tools [9].

Nonetheless, despite the advancements in predicting anxiety disorders, there remains a dearth of research specifically focusing on the application of machine learning for anxiety prediction among individuals with diabetes [12]. Notably, a recent study has emerged with the objective of developing a machine learning model that predicts mental health risk in diabetic patients [13]. This model incorporates crucial data such as demographics, glucometer data, and coaching information [13]. The emerging interest in exploring the intersection of machine learning and anxiety prediction in the context of diabetes underscores the growing importance of further investigations in this field.

## METHOD AND MATERIALS

The proposed methodology in this study can be summarized in 3 steps: Data preparation, machine learning training and machine learning testing. Figure 1 demonstrates the data analysis framework for the predictability steps. Below, we provide further details about various components of the methodological framework.

**Figure:**
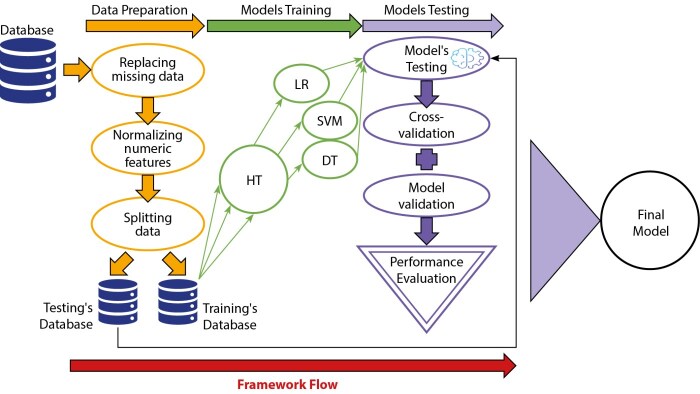
Figure 1: The methodological framework used in this study. HT: Hyperparameters Tuning; LR: Logistic Regression; SVM: Support Vector MachineDT: Decision Tree.

## 1) Data Source

The dataset used was extracted from a previous cross-sectional study conducted at Hassan II University-Hospital of Fez, Morroco, between 2019 and 2020 [3]. This study estimated the prevalence of anxiety and depression in a total of 243 patients with diabetes mellitus and identified their determinants in this region [3].There were 72 cases of anxiety.

## 2) Attributes Description

Predictive models can offer the most value when they are able to support real-time clinical decision making. To maintain the feasibility of applying the algorithms during admission to an acute healthcare setting, we included patient data readily available to clinicians (eg, age, sex, marital status, social insurance, diabetes types, duration of progression, treatment protocol, number of comorbidities, and glycated hemoglobin (HBA1C)) to highlight the sustainability of screening and to aid in real-time proactive decision making.

For the assessment of anxiety status (Anxiety), the Hospital Anxiety and Depression Scale (HADS) [14], validated in Moroccan context [15], was used. It is a self-report scale that identifies anxiety and depressive disorders. It has 14 items. For each item, the response is scored from 0 to 3 on a scale according to the intensity of the symptom during the past week. For the anxiety subscale, threshold values were determined: 0–7: normal; >7 anxiety symptoms [14–15]. This anxiety variable was used as a qualitative variable where patients without anxiety took 0 as the score and anxious patients took 1.

Table I shows the description of these attributes and their values.

**Table:** Table I. Description of selected attributes and their values

Attributes	Description	N (%)
Age in Years (Mean±Standard Error)		48.07±14.25
Gender	Male	102 (42%)
Female	141 (58%)
Marital Status	Single	56 (24.5%)
Married	146 (63.8%)
Divorced or Widowed	27 (11.8%)
Social Insurance	Without	88 (37.4%)
With	147 (62.6%)
Diabetes Types	Type1	67 (27.6%)
Type2	176 (72.4%)
Duration of evolution in Years (Median (Range))		3 (30)
Therapeutic Protocol	Oral Antidiabetics	124 (51%)
Insulin	36 (14.8%)
Both	83 (34.2%)
Number of comorbidities (Median (Range))		0 (3)
Glycated hemoglobin hba1c in numbers (HBA1C) (Mean±Standard Error)		10.05±2.80

## 3) Data Preprocessing

3-1- Replacing missing data.

In this study the Data sets contained some attributes with missing values. Features with more than a 50% missing values threshold will be removed [16].

In view to deal with these missing values, we used an imputation technique as for quantitative variable, especially “HBA1C”, missing values were replaced by the mean of the non-missing values in this column.

For the qualitative variables, we replaced them by the most frequent value.

3-2- Normalizing numeric features.

The MinMaxScaler from sklearn preprocessing was used to normalize numerical variables. This method scales the data to a range between 0 and 1, ensuring that no feature dominates the model due to its scale.

3-3- Splitting data.

Train_test_split function in the Sklearn model selection was used to devise database randomly into two subsets, training data (67%) and testing data (33%).

## 4) Algorithms

In this study, three supervised machine learning models were employed:

## 5) Hyperparameters tuning (HT) : “GridSearchCV”

Grid search is an approach to parameter tuning that methodically builds and evaluates a model for each combination of algorithm parameters specified in a grid [20].

## 6) Cross-validation

A 5-fold cross-validation approach was employed. The dataset was divided into 5 subsets, with each subset serving as the validation set once while the remaining subsets form the training set. This process ensures that the model is evaluated comprehensively [21].

## 7) Model measurments

In order to evaluate the predictive model, various measurements can be calculated such as accuracy, precision, and sensitivity as the following:

## 8) Ethical approval

This study received ethical approval from the Ethics Committee of the Hassan II University Hospital in Fez. All subjects were informed of the study conditions and provided written informed consent. Anonymity and confidentiality were strictly maintained.

## RESULTS

Within the dataset under examination, missing values were detected across some features such as Marital Status (5.76%), Social Insurance (3.29%), and HBA1C (2.47%). Importantly, none of the features had more than 50% missing values. Therefore, all features were retained for subsequent analysis, ensuring the integrity of the dataset.

An evaluation of feature importance association with the target variable was conducted. This analysis is depicted in Figure 2, enumerating attributes from the most significant (Evolution Duration and Gender) to the least (Therapeutic Protocol).

**Figure:**
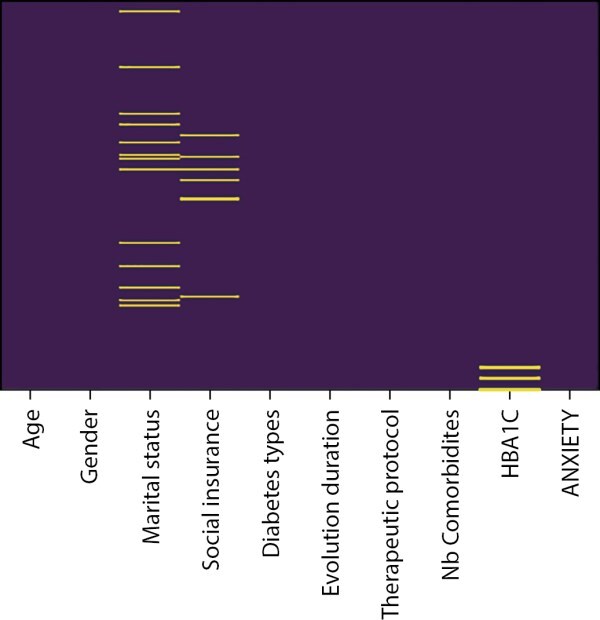
Figure 2: Diagram illustrating the presence of missing values in yellow lines for each variable. Nb Comorbidities: Number of comorbidities.

The obtained results were encouraging in the classification of potentially anxious patients, with the DT algorithm achieving the highest accuracy of 90%, followed by SVM with 66% accuracy, and LR with 63% accuracy. In clinical terms, this means that the DT algorithm correctly identified 90% of patients with anxiety, while SVM and LR correctly identified 66% and 63%, respectively.

The evaluation of the results involved utilizing measures such as accuracy, precision, sensitivity, and the ROC curve, to assess and compare the performance of different algorithms. The findings, as depicted in Table II and Figure 3, highlight the DT algorithm’s superior performance in distinguishing between anxious and non-anxious patients, followed by SVM and LR, respectively. This data is clinically relevant for endocrinologists as it suggests that using the DT algorithm can significantly enhance the accurate identification of patients with anxiety, facilitating timely and effective intervention and management.

**Table:** Table II. Performance comparison of LR, SVM and DT algorithms

Algorithm	Accuracy(Testing)	Sensitivity	Precision	ROC
LR	61%	56%	41%	60%
SVM	69%	76%	50%	71%
DT	96%	96%	92%	96%

**Figure:**
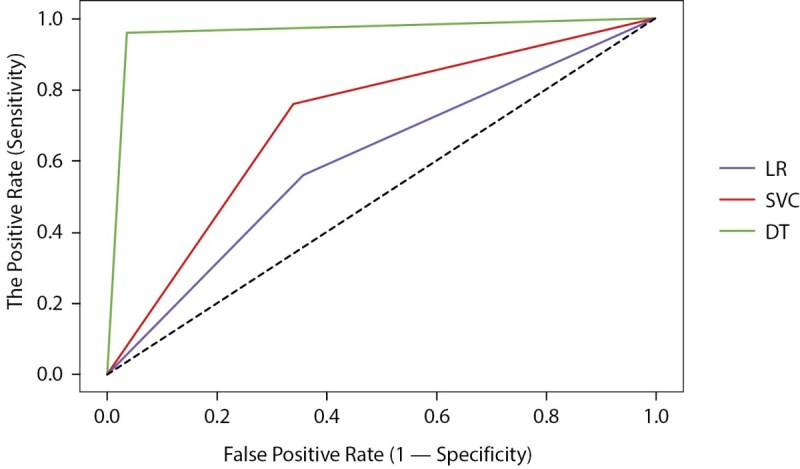
Figure 3: Comparison of different models ROC curves.

## DISCUSSION

This study marks an important contribution to the field by emphasizing the importance of developing a machine learning model specifically tailored for predicting anxiety in Moroccan individuals with diabetes. Anxiety is a prevalent psychological condition that significantly impacts the well-being and quality of life of individuals with diabetes [1-6]. By accurately predicting anxiety, healthcare professionals can implement timely interventions and personalized management strategies to alleviate its impact and improve overall patient outcomes [7–8].

The use of LR, SVM, and DT machine learning algorithms in this study yielded promising results in terms of predicting anxiety in diabetics. These algorithms demonstrated optimal performance, suggesting their potential utility in real-world applications for anxiety prediction. However, it is important to acknowledge the limitations of this study, particularly the use of a small database that was not specifically designed for this research. The small dataset may have affected the generalizability and robustness of the model, potentially leading to biased or less accurate predictions. To address these limitations, the use of a larger dataset is recommended.

Having a large number of variables can increase the risk of overfitting because the model may become too complex, capturing noise and random fluctuations in the training data rather than the underlying patterns [22]. This can lead to excellent performance on the training data but poor generalization to new, unseen data [22]. To avoid this complexity that can led the model to make overly specific predictions that fail to perform well in practical applications, the selection of clinical and laboratory parameters presented in Table 1 was based on their relevance to both diabetes and anxiety, as supported by existing literature [3–6].

Although in this study there were some features more correlated to the target variable, age, gender, marital status, social insurance status, diabetes type, duration of diabetes, therapeutic protocol, number of comorbidities, and glycated hemoglobin (HbA1c) levels are all factors that have been shown to influence both diabetes management and psychological well-being [3–6]. These parameters were chosen based on their established relevance to provide a comprehensive understanding of the patient’s health status and its potential impact on anxiety levels [3–6].

To improve anxiety management in individuals with diabetes, implementing an e-framework based on this predictive model and integrating it into routine clinical practice is highly recommended. Studies have shown that predictive models can be effective tools in improving patient outcomes through early identification of at-risk individuals [23] .A mobile framework would provide an easy-to-access tool and user-friendly interface for healthcare professionals to input patient data and receive accurate risk assessments for anxiety. While the HADS scale has been effectively used in Morocco [15], it requires repeated administration and reevaluation at each clinical consultation, which may not capture early or subtle signs of anxiety [24]. In this study, the machine learning model is not intended only for the early diagnosis of anxiety, rather, it is designed to predict the risk of developing anxiety in diabetic patients in the future. Machine learning models have been increasingly recognized for their ability to predict mental health outcomes by analyzing large, complex datasets [25]. Unlike traditional methods, which may miss subtle psychological symptoms during routine consultations, machine learning models can continuously analyze a broad range of patient-specific data. This allows for more personalized, dynamic insights that could identify at-risk individuals who may not show obvious signs of anxiety in a standard clinical setting. Although the model may not be more cost-effective, its ability to deliver real-time, tailored predictions can enhance the overall accuracy of risk assessment [26] and lead to more personalized treatment strategies for better management of both physical and mental health in diabetic patients.

The essence of the DT model lies in its simplicity and interpretability. DT models are frequently used in clinical decision-making due to their clarity and ease of use [23]. A practitioner can use the DT model by inputting patient data into a software application designed for this purpose. The data entry process is straightforward and typically takes a few minutes, after which the model provides an anxiety prediction. The DT algorithm was the most performant in generating clear and valuable information. Unlike common diagnostic scales for anxiety, the DT model can integrate various clinical parameters and provide a holistic assessment of the patient’s risk [27]. This approach not only supports personalized care but also facilitates continuous monitoring and adjustment of treatment plans based on the patient’s evolving health status.

While the results are promising, it is crucial to consider their implications in a clinical context. The integration of these ML models has the potential to improve the diagnosis and management of anxiety in diabetic patients by enabling early identification and intervention. However, these models have not yet been extensively tested in clinical practice. Future studies should validate their effectiveness and utility in real-world settings, assess their impact on patient outcomes, ease of use for healthcare providers, and overall integration into existing clinical workflows. This validation is essential to ensure the models are both theoretically sound and practically beneficial in improving the quality of care for individuals with diabetes.

## CONCLUSION

In summary, this study introduces a valuable machine learning model for anxiety prediction in Moroccan diabetics, demonstrating the superiority of the decision tree (DT) algorithm over logistic regression (LR) and support vector machine (SVM) algorithms. The development of a mobile framework application based on the DT model is recommended to facilitate its use in daily clinical practice. By integrating this advanced predictive tool into endocrinological practice, healthcare providers can address both physical and mental health aspects, thereby enhancing patient outcomes and overall quality of life.

## ADDITIONAL INFORMATION

Funding. No funding. Conflicts of Interest. The author declares no obvious and potential conflicts of interest related to the content of this article Acknowledgments. The author thanks Fatima Sergeevna Datieva for helpful suggestions and critical reading of the manuscript.

## References

[cit1] Saeedi P, Petersohn I, Salpea P, Malanda B, Karuranga S, Unwin N, Colagiuri S, Guariguata L, Motala AA, Ogurtsova K, Shaw JE, Bright D, Williams R, IDF Diabetes Atlas Committee. Global and regional diabetes prevalence estimates for 2019 and projections for 2030 and 2045: Results from the International Diabetes Federation Diabetes Atlas, 9th edition. Diabetes Res Clin Pract. nov 2019;157:107843. 10.1016/j.diabres.2019.107843 31518657

[cit2] Ministère de la santé du Royaume du Maroc. Rapport de l’enquête nationale sur les facteurs de risque communs des maladies non transmissibles, Steps, 2017-2018 [Internet]. [cité 29 juill 2022]. Disponible sur: https://www.sante.gov.ma/Documents/2019/05/Rapport%20de%20l%20enqu%C3%AAte%20Stepwise.pdf

[cit3] Benmaamar S, Lazar N, El Harch I, Maiouak M, Qarmiche N, Otmani N, Salhi H, Tachfouti N, El Ouahabi H, El Fakir S. Depression and anxiety in patients with diabetes in a Moroccan region. Encephale. 12 oct 2021;S0013-7006(21)00186-X. 10.1016/j.encep.2021.06.014 34654567

[cit4] Smith KJ, Schmitz N. Association of Depression and Anxiety Symptoms With Functional Disability and Disability Days in a Community Sample With Type 2 Diabetes. Psychosomatics. 1 nov 2014;55(6):659-67 25497504 10.1016/j.psym.2014.05.015

[cit5] Huang CJ, Hsieh HM, Tu HP, Jiang HJ, Wang PW, Lin CH. Generalized anxiety disorder in type 2 diabetes mellitus: prevalence and clinical characteristics. Braz J Psychiatry. 17 avr 2020;42(6):621-9. 32321059 10.1590/1516-4446-2019-0605 PMC7678902

[cit6] Park HS, Cho Y, Seo DH, Ahn SH, Hong S, Suh YJ, Chon S, Woo JT, Baik SH, Lee KW, Kim SH. Impact of diabetes distress on glycemic control and diabetic complications in type 2 diabetes mellitus. Sci Rep. 6 mars 2024;14:5568. 10.1038/s41598-024-55901-0 PMC10917807 38448443

[cit7] Gomathy CK. The prediction of disease using machine learning. International Journal of Scientific Research in Engineering and Management (IJSREM). 31 déc 2021.

[cit8] Topol EJ. High-performance medicine: the convergence of human and artificial intelligence. Nat Med. janv 2019;25(1):44-56. 30617339 10.1038/s41591-018-0300-7

[cit9] Pintelas Emmanuel G., Kotsilieris Theodore, Livieris Ioannis E., Pintelas Panagiotis (2019). A review of machine learning prediction methods for anxiety disorders. Proceedings of the 8th International Conference on Software Development and Technologies for Enhancing Accessibility and Fighting Info-exclusion.

[cit10] Kumar P. Assessment of Anxiety, Depression and Stress using Machine Learning Models. Procedia Computer Science. 1 janv 2020;171:1989-98.

[cit11] Nemesure MD, Heinz MV, Huang R, Jacobson NC. Predictive modeling of depression and anxiety using electronic health records and a novel machine learning approach with artificial intelligence. Sci Rep. 21 janv 2021;11(1):1980. 10.1038/s41598-021-81368-4 PMC7820000 33479383

[cit12] Bourkhime H, Qarmiche N, Bahra N, Omari M, Chakri I, Berraho M, Tachfouti N, Fakir SEL, Otmani N. Classification of Depression, Anxiety, and Quality of Life in Diabetic Patients with Machine Learning: Systematic Review. In: Farhaoui Y, Hussain A, Saba T, Taherdoost H, Verma A, éditeurs. Artificial Intelligence, Data Science and Applications. Cham: Springer Nature Switzerland; 2024. p. 263-70. (Lecture Notes in Networks and Systems).

[cit13] Yu J, Chiu C, Wang Y, Dzubur E, Lu W, Hoffman J. A Machine Learning Approach to Passively Informed Prediction of Mental Health Risk in People with Diabetes: Retrospective Case-Control Analysis. Journal of Medical Internet Research. 27 août 2021;23(8):e27709. 10.2196/27709 PMC8433872 34448707

[cit14] Zigmond AS, Snaith RP. The hospital anxiety and depression scale. Acta Psychiatr Scand. juin 1983;67(6):361-70. 6880820 10.1111/j.1600-0447.1983.tb09716.x

[cit15] Bendahhou K, Serhir Z, Ibrahim Khalil A, Radallah D, Amegrissi S, Battas O, Benider A. Validation de la version dialectale Marocaine de l’échelle « HADS ». Revue d’Épidémiologie et de Santé Publique. 1 mai 2017;65:S53.

[cit16] https://cran.r-project.org/web/packages/missCompare/vignettes/misscompare.html

[cit17] Subasi A. Machine learning techniques. In: Practical Machine Learning for Data Analysis Using Python [Internet]. Elsevier; 2020 [cité 28 juill 2022]. p. 91 202. Disponible sur: https://linkinghub.elsevier.com/retrieve/pii/B9780128213797000035

[cit18] Thorsten J. Making Large-Scale SVM Learning Practical. Advances in Kernel Methods - Support Vector Learning, Bernhard Scholkopf, Christopher J. C. Burges, and Alexander J. Smola (eds.), MIT Press, Cambridge, USA, 1998. Disponible sur: https://www.cs.cornell.edu/people/tj/publications/joachims_99a.pdf

[cit19] Navada A, Ansari A, Patil S, Sonkamble B. Overview of use of decision tree algorithms in machine learning. Proceedings - 2011 IEEE Control and System Graduate Research Colloquium, ICSGRC 2011. 2011. 37 p.

[cit20] Padierna L, Carpio M, Rojas-Dominguez A, Soberanes H, Baltazar R, Fraire-Huacuja H. Hyper-Parameter Tuning for Support Vector Machines by Estimation of Distribution Algorithms. In 2017. p. 787-800.

[cit21] Berrar D. Cross-Validation. Encyclopedia of Bioinformatics and Computational Biology, Volume 1, Elsevier, pp. 542–545. In 2018. https://doi.org/10.1016/B978-0-12-809633-8.20349-X1

[cit22] Van der Schaaf A, Xu CJ, van Luijk P, van’t Veld AA, Langendijk JA, Schilstra C. Multivariate modeling of complications with data driven variable selection: Guarding against overfitting and effects of data set size. Radiotherapy and Oncology. 1 oct 2012;105(1):115-21. 10.1016/j.radonc.2011.12.006 22264894

[cit23] Toma M, Wei OC. Predictive Modeling in Medicine. Encyclopedia. juin 2023;3(2):590-601.

[cit24] Bjelland I, Dahl AA, Haug TT, Neckelmann D. The validity of the Hospital Anxiety and Depression Scale: An updated literature review. Journal of Psychosomatic Research. 1 févr 2002;52(2):69-77. 11832252 10.1016/s0022-3999(01)00296-3

[cit25] Shatte ABR, Hutchinson DM, Teague SJ. Machine learning in mental health: a scoping review of methods and applications. Psychol Med. juill 2019;49(9):1426-48. 30744717 10.1017/S0033291719000151

[cit26] Topol EJ. High-performance medicine: the convergence of human and artificial intelligence. Nat Med. janv 2019;25(1):44-56. 30617339 10.1038/s41591-018-0300-7

[cit27] Breiman L, Friedman J, Olshen RA, Stone CJ. Classification and Regression Trees. New York: Chapman and Hall/CRC; 2017. 368 p.

